# Supplementary material for: Risk of acute kidney injury associated with anti-pseudomonal and anti-MRSA antibiotic strategies in critically ill patients
Source: PLoS One. 2022 Mar 10;17(3):e0264281. doi: 10.1371/journal.pone.0264281 (PMC8912201; doi:10.1371/journal.pone.0264281)
Supplement: S1 Fig — (PDF) [file pone.0264281.s001.pdf]

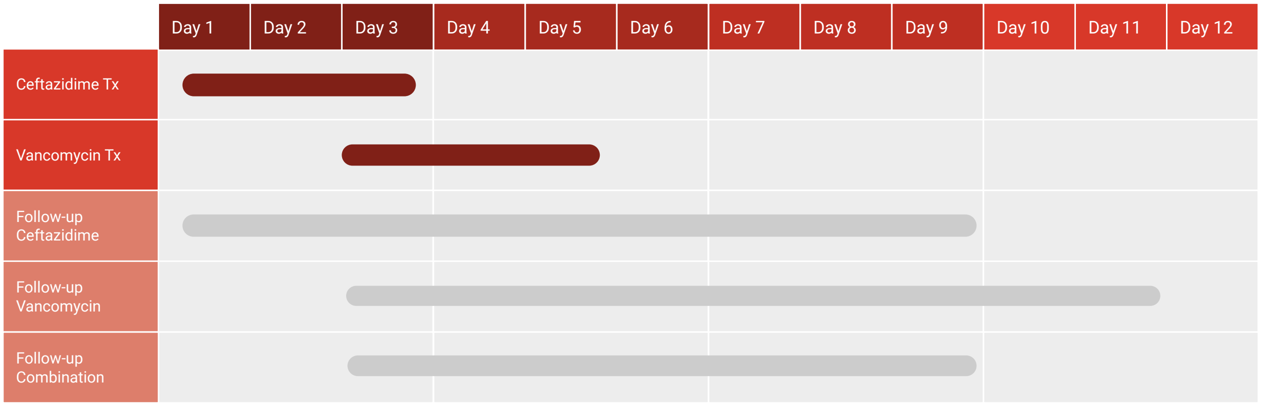

S1 Fig. Example of the follow-up for the primary endpoint (new or worsening AKI within 7 days)
Using data from a hypothetical patient receiving ceftazidime for 3 consecutive days, vancomycin plus ceftazidime for 24h, then vancomycin only for 2 days. The concomitant presence of another antibiotic was adjusted in the multivariate model.
