# Supplementary material for: Risk of acute kidney injury associated with anti-pseudomonal and anti-MRSA antibiotic strategies in critically ill patients
Source: PLoS One. 2022 Mar 10;17(3):e0264281. doi: 10.1371/journal.pone.0264281 (PMC8912201; doi:10.1371/journal.pone.0264281)
Supplement: S1 Table — (PDF) [file pone.0264281.s002.pdf]

S1 Table. Absolute risk of major clinical endpoints for each antibiotic class received by admitted patients, n (%)

	**Anti-pseudomonas** (n=13,255)	**Anti-MRSA** (n=16,424)	**Anti-pseudomonas + Anti-MRSA** (n=10,756)
Acute kidney injury events			
New or worsening AKI *(from any date of antibiotic therapy duration)†*			
Within 7 days	7,578 (57)	9,672 (59)	6,646 (62)
New onset KRT *(from any date of antibiotic therapy)*			
Within 7 days	595 (4.5)	637 (3.9)	570 (5.3)
Within 30 days	599 (4.5)	642 (3.9)	574 (5.3)
Maximum AKI stage achieved *(from any date of antibiotic therapy duration)†*			
Within 7 days			
Stage 1	4,275 (32)	5,895 (36)	3,461 (32)
Stage 2	2,762 (21)	3,129 (19)	2,411 (22)
Stage 3 or KRT	2,835 (21)	3,064 (19)	2,586 (24)
Mortality events			
In-hospital mortality	2,479 (19)	2,672 (16)	2,307 (21)

AKI; Acute kidney injury, MRSA; Methicillin-resistant staphylococcus aureus, KRT; Kidney replacement therapy

† Considering the highest available creatinine measurement within 48-h and 7 days after any treatment day for each patient, based on KDIGO criteria.
