# Supplementary material for: Risk of acute kidney injury associated with anti-pseudomonal and anti-MRSA antibiotic strategies in critically ill patients
Source: PLoS One. 2022 Mar 10;17(3):e0264281. doi: 10.1371/journal.pone.0264281 (PMC8912201; doi:10.1371/journal.pone.0264281)
Supplement: S2 Table — (PDF) [file pone.0264281.s003.pdf]

Table S2. Absolute risk of major clinical endpoints for each antibiotic class received by observation periods, n (%)

	**Anti-pseudomonas** (n=118,200)	**Anti-MRSA** (n=130,123)	**Anti-pseudomonas + Anti-MRSA** (n=78,357)
Acute kidney injury events			
New or worsening AKI†			
Within 7 days	33,468 (28)	39,707 (31)	23,815 (30)
New onset KRT			
Within 7 days	2,088 (1.8)	2,190 (1.7)	1,625 (2.1)
Within 30 days	3,566 (3.0)	3,657 (2.8)	2,644 (3.4)
Maximum AKI stage†			
Within 7 days			
Stage 1	32,933 (28)	39,155 (30)	22,267 (28)
Stage 2	22,595 (19)	23,655 (18)	15,712 (20)
Stage 3 or KRT	21,590 (18)	22,886 (18)	15,769 (20)
Mortality events			
Within 7 days	10,000 (8.5)	10,353 (8.0)	7,955 (10)
Within 30 days	20,929 (18)	21,185 (16)	16,114 (21)

AKI; Acute kidney injury, MRSA; Methicillin-resistant staphylococcus aureus, KRT; Kidney replacement therapy
† Based on KDIGO criteria.
