# Supplementary material for: Risk of acute kidney injury associated with anti-pseudomonal and anti-MRSA antibiotic strategies in critically ill patients
Source: PLoS One. 2022 Mar 10;17(3):e0264281. doi: 10.1371/journal.pone.0264281 (PMC8912201; doi:10.1371/journal.pone.0264281)
Supplement: S3 Table — (PDF) [file pone.0264281.s004.pdf]

Table S3. Risk of new or worsening AKI and KRT associated with exposure to various anti-pseudomonas, anti-MRSA or their combination (univariate)

	**Observation days†**		**AKI within 7d**, OR [95% CI]	**New onset KRT within 7d**, OR [95% CI]	**New onset KRT within 30d**, OR [95% CI]
	**Investigated drug**	**Comparison**			
**Anti-pseudomonas** (REF = PTZ)					
Non-PTZ anti-pseudomonas (any)	73,544	32,648	0.83 [0.78-0.89]***	0.72 [0.57-0.90]**	0.64 [0.48-0.86]**
Ciprofloxacin	25,896	35,387	0.82 [0.76-0.89]***	0.62 [0.46-0.84]**	0.80 [0.55-1.17]^{NS}
Aminoglycoside	7,365	37,975	1.30 [1.17-1.46]***	0.75 [0.50-1.13]^{NS}	0.68 [0.49-0.94]*
Ceftazidime	6,678	38,964	0.85 [0.75-0.96]*	0.87 [0.61-1.23]^{NS}	0.82 [0.45-1.50]^{NS}
Cefepime	19,705	38,470	0.92 [0.85-1.00]^{NS}	0.74 [0.56-0.98]*	0.66 [0.39-1.13]^{NS}
Carbapenem	19,259	38,270	0.69 [0.63-0.76]***	0.67 [0.48-0.94]*	0.41 [0.22-0.76]**
Aztreonam	3,501	39,168	0.77 [0.64-0.91]**	1.02 [0.62-1.70]^{NS}	1.49 [0.59-3.78]^{NS}
**Anti-MRSA** (REF = vancomycin)					
Non-vanco anti-MRSA (any)	10,474	112,938	0.74 [0.66-0.83]***	1.27 [0.81-1.99]^{NS}	0.33 [0.08-1.38]^{NS}
Daptomycin	3,512	113,882	0.76 [0.63-0.92]**	1.09 [0.49-2.43]^{NS}	0.30 [0.02-4.61]^{NS}
Linezolid	7,151	113,415	0.74 [0.65-0.85]***	1.34 [0.81-2.22]^{NS}	0.32 [0.06-1.70]^{NS}
**Anti-pseudomonas + Anti-MRSA** (REF = PTZ + vanco)					
Non-PTZ anti-pseudomonas + non-vanco anti-MRSA (any)	6,471	22,873	0.71 [0.61-0.82]***	1.20 [0.73-1.99]^{NS}	0.45 [0.12-1.69]^{NS}
Ciprofloxacin + Daptomycin	391	24,934	0.73 [0.45-1.17]^{NS}	0.59 [0.15-2.34]^{NS}	0.69 [0.22-2.19]^{NS}
Ciprofloxacin + Linezolid	574	24,783	0.61 [0.41-0.91]*	1.04 [0.47-2.32]^{NS}	0.91 [0.56-1.49]^{NS}
Aminoglycoside + Daptomycin	177	27,094	1.65 [0.95-2.88]^{NS}	0.87 [0.28-2.67]^{NS}	0.95 [0.44-2.08]^{NS}
Aminoglycoside + Linezolid	382	26,942	1.12 [0.74-1.70]^{NS}	2.89 [0.96-8.70]^{NS}	NS‡
Ceftazidime + Daptomycin	59	27,656	0.49 [0.07-2.83]^{NS}	2.39 [0.46-12.53]^{NS}	1.43 [0.64-3.19]^{NS}
Ceftazidime + Linezolid	278	27,501	0.88 [0.60-1.30]^{NS}	NS‡	NS‡
Cefepime + Daptomycin	439	27,225	0.89 [0.57-1.38]^{NS}	1.80 [0.71-4.61]^{NS}	1.80 [1.09-2.97]*
Cefepime + Linezolid	514	27,065	0.76 [0.51-1.14]^{NS}	0.55 [0.09-3.39]^{NS}	NS‡
Carbapenem + Daptomycin	995	27,158	0.76 [0.56-1.04]^{NS}	0.50 [0.11-2.34]^{NS}	NS‡
Carbapenem + Linezolid	1854	27,036	0.83 [0.65-1.06]^{NS}	1.89 [0.97-3.68]^{NS}	0.72 [0.10-5.18]^{NS}
Aztreonam + Daptomycin	207	27,835	1.50 [0.81-2.76]^{NS}	0.49 [0.10-2.29]^{NS}	1.02 [0.46-2.24]^{NS}
Aztreonam + Linezolid	250	27,677	0.33 [0.20-0.55]***	0.71 [0.12-4.16]^{NS}	1.06 [0.44-2.56]^{NS}

*^{NS}: p-value≥.05, *: p-value<.05, **: p-value<.01, ***: p-value<.001, AKI: Acute kidney injury, KRT: Kidney replacement therapy, REF: Reference group, PTZ: Piperacillin-tazobactam*

Results reported are Odds ratios with confidence intervals from a generalized estimating equation (binomial GEE) non-adjusted.

†Observations where both investigated, and comparator antibiotics were concomitantly received and where KRT was ongoing (ie. not at risk of progression) were excluded from the analysis.

‡ Results (OR) very close to 1 with limited event rates, no valid confidence interval can be inferred using the current GEE analysis.
