# Supplementary material for: Risk of acute kidney injury associated with anti-pseudomonal and anti-MRSA antibiotic strategies in critically ill patients
Source: PLoS One. 2022 Mar 10;17(3):e0264281. doi: 10.1371/journal.pone.0264281 (PMC8912201; doi:10.1371/journal.pone.0264281)
Supplement: S4 Table — (PDF) [file pone.0264281.s005.pdf]

Table S4. Risk of new or worsening AKI and KRT associated with exposure to various anti-pseudomonas, anti-MRSA or their combination (entire cohort and for at least 72h) (multivariate)

	**Observation days**[†]		**AKI within 7d**, OR [95% CI]	**New onset KRT within 7d**, OR [95% CI]	**New onset KRT within 30d**, OR [95% CI]
	**Investi-gated drug**	**Compa-rison**			
**Non-PTZ anti-pseudomonas** (REF = PTZ)	73,544	32,648	0.85 [0.80-0.91]***	0.86 [0.69-1.07]^{NS}	0.72 [0.57-0.90]**
With ATB tx duration ≥72h	65,144	29,555	0.84 [0.78-0.90]***	0.87 [0.70-1.10]^{NS}	0.72 [0.58—0.90]**
**Non-vanco anti-MRSA** (REF = vancomycin)	10,474	112,938	0.71 [0.64-0.80]***	1.13 [0.80-1.60]^{NS}	0.58 [0.40-0.85]**
With ATB tx duration ≥72h	10,131	88,636	0.73 [0.65-0.82]***	1.12 [0.79-1.58]^{NS}	0.56 [0.37-0.84]**
**Non-PTZ anti-pseudomonas + non-vanco anti-MRSA** (REF = PTZ + vancomycin)	6,471	22,873	0.63 [0.54-0.73]***	1.05 [0.61-1.79]^{NS}	0.51 [0.26-1.01]^{NS}
With ATB tx duration ≥72h	6,285	20,683	0.64 [0.55-0.74]***	1.05 [0.61-1.80]^{NS}	0.51 [0.25-1.01]^{NS}

*^{NS} : p-value≥.05, * : p-value<.05, **: p-value<.01, ***: p-value<.001, AKI: Acute kidney injury, KRT: Kidney replacement therapy, REF: Reference group, PTZ: Piperacillin-tazobactam*

Results reported are Odds ratios with confidence intervals from a generalized estimating equation (binomial GEE) adjusted for: Age, sex, ethnicity, comorbidities (heart failure, liver disease and diabetes), SOFA score, hyperlactatemia, vasopressors, chronic kidney disease, antibiotic treatment duration, active bacteremia, positive ventilation, active corticosteroid therapy and leukopenia. Analyses for all anti-pseudomonal agents were also adjusted for the presence of a concomitant anti-MRSA agent, while analyses for anti-MRSA agents were adjusted for the presence of an anti-pseudomonal agent.

[†]Observations where both investigated, and comparator antibiotics were concomitantly received and where KRT was ongoing (ie. not at risk of progression) were excluded from the analysis.
