# Supplementary material for: Risk of acute kidney injury associated with anti-pseudomonal and anti-MRSA antibiotic strategies in critically ill patients
Source: PLoS One. 2022 Mar 10;17(3):e0264281. doi: 10.1371/journal.pone.0264281 (PMC8912201; doi:10.1371/journal.pone.0264281)
Supplement: S5 Table — (PDF) [file pone.0264281.s006.pdf]

**Table S5. Risk of new or worsening AKI associated with exposure to various anti-pseudomonas, anti-MRSA or their combination (subgroup analyses, multivariate)**			
	**Observation days[†]**		**AKI within 7d**, OR [95% CI]
	**Investigated drug**	**Comparison**	
*Patients with **confirmed pseudomonas** spp. infection or colonization*			
**Non-PTZ anti-pseudomonas** (REF = PTZ)	12,394	4,050	0.93 [0.78-1.11][NS]
**Non-vanco anti-MRSA** (REF = Vancomycin)	2,411	11,730	0.76 [0.59-0.98][*]
**Non-PTZ anti-pseudomonas + non-vanco anti-MRSA** (REF = PTZ+Vancomycin)	1,632	2,307	0.69 [0.50-0.95][*]
*Patients with **confirmed MRSA** infection or colonization*			
**Non-PTZ anti-pseudomonas** (REF = PTZ)	11,427	5,159	0.83 [0.70-0.99][*]
**Non-vanco anti-MRSA** (REF = Vancomycin)	2,901	22,402	0.77 [0.62-0.96][*]
**Non-PTZ anti-pseudomonas + non-vanco anti-MRSA** (REF = PTZ+Vancomycin)	1,500	3,812	0.66 [0.48-0.90][**]
*Patients with **CKD***			
**Non-PTZ anti-pseudomonas** (REF = PTZ)	11,194	6,312	0.91 [0.78-1.05][NS]
**Non-vanco anti-MRSA** (REF = Vancomycin)	1,327	20,173	0.85 [0.63-1.15][NS]
**Non-PTZ anti-pseudomonas + non-vanco anti-MRSA** (REF = PTZ+Vancomycin)	719	4,418	0.72 [0.47-1.10][NS]
*Patients with antibiotic **treatment initiated within the first 48h** of ICU admission*			
**Non-PTZ anti-pseudomonas** (REF = PTZ)	38,790	18,762	0.82 [0.76-0.90][***]
**Non-vanco anti-MRSA** (REF = Vancomycin)	5,314	62,715	0.75 [0.64-0.88][***]
**Non-PTZ anti-pseudomonas + non-vanco anti-MRSA** (REF = PTZ+Vancomycin)	3,629	13,659	0.65 [0.54-0.78][***]
*Patients with antibiotic **treatment initiated after 72h** of ICU admission time (nosocomial infection)*			
**Non-PTZ anti-pseudomonas** (REF = PTZ)	10,269	5,548	0.90 [0.83-0.97][**]
**Non-vanco anti-MRSA** (REF = Vancomycin)	1,217	17,865	0.69 [0.49-0.95][*]
**Non-PTZ anti-pseudomonas + non-vanco anti-MRSA** (REF = PTZ+Vancomycin)	670	3,911	0.44 [0.27-0.71][***]
*Patients receiving the **same antibiotic drug** for **≥72h***			
**Non-PTZ anti-pseudomonas** (REF = PTZ)	47,808	21,148	0.80 [0.68-0.94][**]
**Non-vanco anti-MRSA** (REF = Vancomycin)	8,404	59,113	0.74 [0.65-0.84][***]
**Non-PTZ anti-pseudomonas + non-vanco anti-MRSA** (REF = PTZ+Vancomycin)	5,442	14,374	0.72 [0.61-0.85][***]
*Patients with **SOFA score** >**5** at ICU admission[‡]*			
**Non-PTZ anti-pseudomonas** (REF = PTZ)	29,838	16,168	0.85 [0.78-0.93][***]
**Non-vanco anti-MRSA** (REF = Vancomycin)	4,585	52,250	0.78 [0.66-0.93][**]
**Non-PTZ anti-pseudomonas + non-vanco anti-MRSA** (REF = PTZ+Vancomycin)	3,081	11,564	0.68 [0.55-0.84][***]
***Exclusion** of patients receiving an **aminoglycoside** agent*			
**Non-PTZ anti-pseudomonas** (REF = PTZ)	66,179	32,648	0.81 [0.76-0.87][***]
**Non-vanco anti-MRSA** (REF = Vancomycin)	9,817	108,413	0.72 [0.64-0.80][***]
**Non-PTZ anti-pseudomonas + non-vanco anti-MRSA** (REF = PTZ+Vancomycin)	5,929	22,873	0.61 [0.53-0.71][***]

***Exclusion** of patients with known **supratherapeutic vancomycin level** within 48h (>20 mg/L)*			
**Non-PTZ anti-pseudomonas** (REF = PTZ)	63,435	26,933	0.88 [0.82-0.94]***
**Non-vanco anti-MRSA** (REF = Vancomycin)	10,460	88,675	0.71 [0.64-0.80]***
**Non-PTZ anti-pseudomonas + non-vanco anti-MRSA** (REF = PTZ+Vancomycin)	6,461	17,308	0.65 [0.56-0.76]***

*NS : p-value≥.05, * : p-value<.05, **: p-value<.01, ***: p-value<.001 . AKI: Acute kidney injury, KRT: Kidney replacement therapy, REF: Reference group, PTZ: Piperacillin-tazobactam*

Results reported are Odds ratios with confidence intervals from a generalized estimating equation (binomial GEE) adjusted for: Age, sex, ethnicity, comorbidities (heart failure, liver disease and diabetes), SOFA score, hyperlactatemia, vasopressors, chronic kidney disease, antibiotic treatment duration, active bacteremia, positive ventilation, active corticosteroid therapy and leukopenia. Analyses for all anti-pseudomonal agents were also adjusted for the presence of a concomitant anti-MRSA agent, while analyses for anti-MRSA agents were adjusted for the presence of an anti-pseudomonal agent.

†Observations where both investigated, and comparator antibiotics were concomitantly received and where KRT was ongoing (ie. not at risk of progression) were excluded from the analysis

‡ Not adjusted for CKD in the current model

ž Not adjusted for SOFA score in the current model
