# Supplementary material for: Risk of acute kidney injury associated with anti-pseudomonal and anti-MRSA antibiotic strategies in critically ill patients
Source: PLoS One. 2022 Mar 10;17(3):e0264281. doi: 10.1371/journal.pone.0264281 (PMC8912201; doi:10.1371/journal.pone.0264281)
Supplement: S6 Table — (PDF) [file pone.0264281.s007.pdf]

Table S6. Change in serum creatinine and urea within 72-h associated with exposure to PTZ compared to another anti-pseudomonas with or without vancomycin

	Observation days†		Creatinine change within 72h		Urea change within 72h	
	**PTZ**	**Compa-rison**	ß estimate of mg/dl difference [95% CI]	ß estimate of % variation [95% CI]	ß estimate of mg/dl difference [95% CI]	ß estimate of % variation [95% CI]
All patients						
Anti-pseudomonas	32,066	71,817	0.05 [0.04; 0.06]***	3.2 [2.3; 4.1]***	-0.01 [-0.28; 0.25]NS	0.2 [-0.9; 1.2]NS
Anti-pseudomonas + vancomycin	22,803	38,911	0.06 [0.04; 0.08]***	3.9 [2.7; 5.1]***	-0.19 [-0.51; 0.12]NS	-0.5 [-1.8; 0.8]NS
Patients with progression to stage 1 AKI within 7 days						
Anti-pseudomonas	10,194	19,931	0.00 [0.00; 0.01]NS	-0.3 [-0.9; 0.3]NS	-0.94 [-1.22; -0.64]***	-3.2 [-4.5; -2.0]***
Anti-pseudomonas + vancomycin	7,399	11,181	0.00 [0.00; 0.01]NS	-0.2 [-0.9; 0.6]NS	-1.24 [-1.59; -0.90]***	-5.0 [-6.5; -3.5]***
Patients with progression to stage 2 or 3 AKI within 7 days						
Anti-pseudomonas	12,409	24,371	0.08 [0.05; 0.10]***	3.8 [1.8; 5.8]***	-0.22 [-0.73; 0.29]NS	0.6 [-1.7; 2.8]NS
Anti-pseudomonas + vancomycin	9,020	13,905	0.09 [0.05; 0.12]***	5.6 [3.1; 8.2]***	-0.24 [-0.82; 0.35]NS	1.3 [-1.3; 3.9]NS

*NS : p-value≥.05, *: p-value<.05, **: p-value<.01, ***: p-value<.001, AKI; Acute kidney injury, PTZ: Piperacillin-tazobactam*
Results reported are beta-estimate from a generalized estimating equations (linear GEE) analysis, the magnitudes observed correlate with change in mg/dL and relative variation (%).
†Observations where both investigated, and comparator antibiotics were concomitantly received and where KRT was ongoing were excluded from the analysis.
